# Cognitive fatigue in individuals with traumatic brain injury is associated with caudate activation

**DOI:** 10.1038/s41598-017-08846-6

**Published:** 2017-08-21

**Authors:** G. R. Wylie, E. Dobryakova, J. DeLuca, N. Chiaravalloti, K. Essad, H. Genova

**Affiliations:** 1 Kessler Foundation, 120 Eagle Rock Avenue, Suite 100, East Hanover, New Jersey 07936 USA; 2 Department of Physical Medicine and Rehabilitation, Rutgers University, New Jersey Medical School, Newark, NJ 07101 USA; 3 The War Related Illness and Injury Study Center, The Department of Veterans’ Affairs, New Jersey Healthcare System, East Orange Campus, East Orange, NJ 07018 USA; 4 Department of Neurology, Rutgers University, New Jersey Medical School, Newark, NJ 07101 USA; 5 Dartmouth College, Dartmouth College Medical School, Hanover, NH 03755 USA

## Abstract

We investigated differences in brain activation associated with cognitive fatigue between persons with traumatic brain injury (TBI) and healthy controls (HCs). Twenty-two participants with moderate-severe TBI and 20 HCs performed four blocks of a difficult working memory task and four blocks of a control task during fMRI imaging. Cognitive fatigue, assessed before and after each block, was used as a covariate to assess fatigue-related brain activation. The TBI group reported more fatigue than the HCs, though their performance was comparable. Regarding brain activation, the TBI group showed a Task X Fatigue interaction in the caudate tail resulting from a positive correlation between fatigue and brain activation for the difficult task and a negative relationship for the control task. The HC group showed the same Task X Fatigue interaction in the caudate head. Because we had prior hypotheses about the caudate, we performed a confirmatory analysis of a separate dataset in which the same subjects performed a processing speed task. A relationship between Fatigue and brain activation was evident in the caudate for this task as well. These results underscore the importance of the caudate nucleus in relation to cognitive fatigue.

## Introduction

Traumatic Brain Injury (TBI) is one of the most prevalent causes of neurological insult in the US, with over 1.7 million cases reported annually^1^, and it is a growing problem in other countries^2^. The effects of TBI on cognition are profound and diverse: TBI has been shown to affect working memory (the ability to hold information ‘in mind’ for short periods of time, and also to manipulate that information) e.g., refs 3–5, processing speed e.g. refs 6 and 7, neural efficiency^4, 5^ and cognitive fatigue e.g. refs 8–11. In the work presented here, we focused on cognitive fatigue, or the fatigue that results from performing cognitive tasks.

Cognitive fatigue can be defined as a subjective lack of mental energy that is perceived by the individual (or caregiver) to interfere with usual and desired activities^12^. It has been known for over a century that individuals who have sustained neurological damage frequently report cognitive fatigue^13^, yet a comprehensive and accurate model for studying fatigue remains elusive. This difficulty stems, at least in part, from the lack of consistent correlation between objective measures of fatigue such as response time (RT), error rate (ER) or even brain lesions^14^ and subjective reports of fatigue^13^. That is, although subjects’ experience of fatigue may increase as a cognitive task is repeatedly performed, performance is not usually affected.

The inability to relate behavioral decrements to self-reported cognitive fatigue led us to investigate other dependent measures that may correlate better with fatigue: specifically, the BOLD signal associated with changes in neural activation^8^. While others have investigated the relationship between fatigue and white matter integrity^15^, we chose fMRI on the hypothesis that increased BOLD activation represented an increase in the cognitive work that individuals with TBI must expend in order to perform a given task^16^. In that work^8^, we showed that individuals with TBI recruit the fronto-striatal network more during performance of a demanding cognitive task compared to healthy participants, including ventromedial prefrontal cortex (VMPFC), parietal areas, and the basal ganglia. This network has been previously shown to be involved in cognitive fatigue^16–19^.

Additionally, cognitive fatigue research has been limited due to the fact that historically, investigators have assessed subjective fatigue using scales which assess “trait” fatigue, rather than “state” fatigue. For example, one frequently used fatigue questionnaire is the Fatigue Severity Scale (FSS), which asks participants to rate their fatigue over the past week. Thus, the FSS is a measure of trait fatigue, or the extent to which subjects are prone to experiencing fatigue over an extended period of time. Recently, the literature on fatigue has begun to distinguish between state and trait fatigue^19–21^. The assessment of state fatigue, or the extent to which subjects are experiencing fatigue at the instant of assessment, may more consistently correlate with behavior inasmuch as it is measured at the same time the behavior is measured. Thus, in the current study, we used the Visual Analog Scale of Fatigue (VAS-F), which asks subjects to rate the amount of fatigue they experience at the time of behavioral performance.

In previous work, we have shown that the caudate nucleus of the basal ganglia shows a different pattern of activation over time in individuals with TBI relative to HCs^8^. This result accorded well with a model of fatigue proposed by Chauduri & Behan^17^ in which the basal ganglia played a key role in the experience of fatigue; however, Kohl *et al*.^8^ did not assess subjects’ instantaneous (state) experience of fatigue, but rather inferred the presence of fatigue based on the pattern of brain activation over time. The current study therefore represents the first investigation of state fatigue in individuals with moderate-severe TBI. Furthermore, we specifically investigated the role of the caudate nucleus in fatigue by investigating whether the caudate modulates its activity in direct proportion to subjects’ instantaneous (state) experience of fatigue^19^. Indeed, while the caudate nucleus, and the striatum as a whole, have been previously considered to be responsible purely for the control of motor behavior^22^, recent evidence from animal and human research demonstrates that this region plays a significant role in a variety of cognitive behaviors, such as learning^23–25^, outcome processing^26, 27^, and working memory^28, 29^. More recent evidence suggests that the fatigue resulting from such cognitive processes may also be reflected in caudate nucleus activation^30^. This is particularly likely in the TBI population given the nature of brain injury sustained by most individuals with TBI: damage to the prefrontal cortex that sends afferent connections to the basal ganglia and interruption of dopamine transmission in the basal ganglia after TBI^31^. Indeed, cognitive fatigue has been associated with a network of areas in the striatum and PFC including vmPFC, nucleus accumbens and anterior cingulate cortex (ACC) in children who have sustained a TBI^32^.

In the current study, we investigated the role of the caudate nucleus in fatigue by asking subjects to perform a working memory task (the N-Back task), since working memory is known to be compromised in TBI. Secondly, we included both a difficult task (the 2-back condition) and a less difficult task (the 0-back condition). We hypothesized that the TBI group would show more fatigue-related activation for both the 0-back task and for the 2-back task than the healthy control (HC) group, and that this would be associated with basal ganglia activation^17^. We then performed a second experiment using a processing speed task to confirm that the activation in the caudate was related to fatigue rather than to the task.

## Experiment 1

### Methods

#### Participants

Twenty-two right-handed participants with moderate to severe TBI (mean age = 41.4 ± 13.2 (standard deviation [S.D.]) years; education = 14.6 ± 1.9 (S.D.) years; mean time since injury = 79.5 ± 51.0 (S.D.) months) and 20 healthy control participants (age = 37.7 ± 10.9 (S.D.) years; education = 15.3 ± 1.8 (S.D.) years) participated in the study. The majority of the individuals in the TBI group had sustained their injury as a result of a motor-vehicle accident (59%), followed by falls (32%), followed by assault (9%). The groups did not differ in age or education. The groups were marginally different in terms of gender (14 men in the TBI group, 8 men in the HC group; **χ** ^2^ (1) = 3.6, p = 0.057). While this was not a robust difference, we nevertheless included gender as a covariate in all analyses. Twenty of the 22 participants in the TBI group had sustained a moderate-to-severe TBI, defined as the lowest Glasgow Coma Scale (GCS) rating in the first 24 hours following injury being below 13^33^. When a GCS score was not available, subjects were included only when there was sufficient medical documentation that allowed for a post-hoc estimation of initial GCS, or if other confirmatory data (e.g., positive anatomic neuroimaging findings, focal neurologic signs) were available. Two subjects in the TBI group were included who were considered to have had complicated mild TBIs. One participant had a GCS of 15, but had sustained a subarachnoid hemorrhage at the time of the accident. The other had a GCS of 14, but had sustained a subdural hematoma, intra-parenchymal hemorrhage, and had cerebral edema as a result of the accident. Because the results did not change when the data from these participants was excluded, their data were retained in the sample.

For both the TBI and HC groups, subjects were: 1) free of a history of prior neurological insult or disease (other than TBI for the TBI group) (e.g., stroke, seizures, or brain tumor); 2) free from significant psychiatric history (such as schizophrenia or bipolar disorder) due to the potential influence of such disorders on cognitive functioning (assessed by self-report corroborated by medical records when available); 3) right handed due to the effect of mixed hand dominance on cerebral organization; 4) free of alcohol or drug abuse history. Subjects currently taking benzodiazepines, neuroleptics, or psycho-stimulants were excluded due to the potential effects of these medications on cognition and the hemodynamic response. For all study participants, additional exclusionary criteria associated with MRI (ferrous metal in the body) were discussed and strictly enforced.

**Figure 1:**
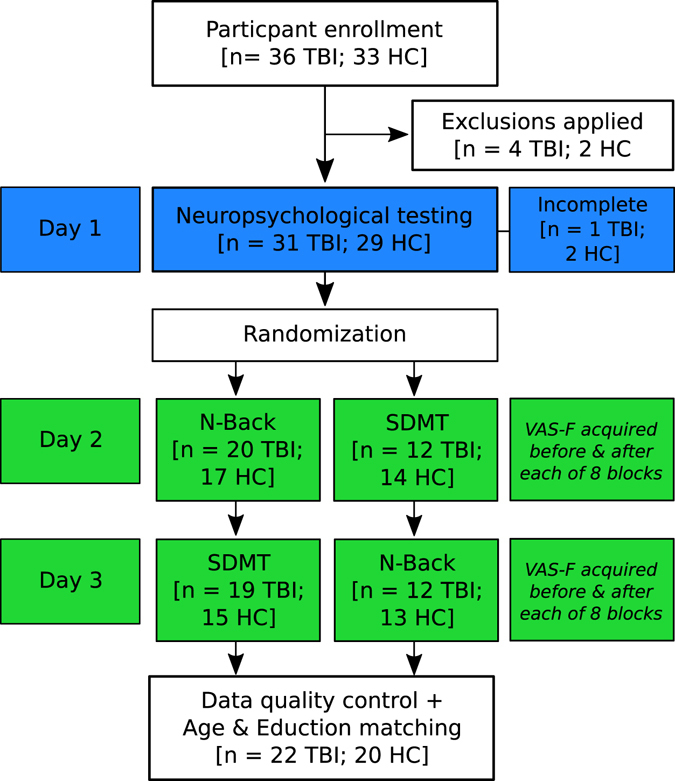
A flow diagram of the experimental design. Thirty-six individuals with TBI and 33 HCs were enrolled. After exclusions were applied (e.g., MRI incompatibility, psychiatric history, claustrophobia), the sample was reduced to 32 individuals with TBI and 31 HCs. Subjects then participated in a day of neuropsychological (NP) testing. Three subjects did not complete the NP test battery (1 TBI, 2 HC), and because the focus of the current study was on functional neuroimaging, these subjects were retained in the sample. Subjects were then randomized into two groups. One group performed the N-Back test while in the fMRI scanner on Day 2 and the SDMT task in the scanner on Day 3. For the other group, the order of N-Back and SDMT tasks was reversed. There was some attrition during this three-day experiment, resulting in a final sample of 31 individuals with TBI and 28 HCs. After applying data quality control (e.g., excluding subjects with excessive motion) and matching the two groups on Age and Education, the final sample was 22 individuals with TBI and 20 HCs.

All subjects participated in two scanning sessions which, for expository purposes, we will refer to as Experiment 1 and 2. In one session, subjects performed the N-Back working memory task (see below for details) and in the other they performed the modified symbol-digit modalities task (SDMT; see Experiment 2). The order in which subjects performed these tasks was counterbalanced (see Fig. 1).

The Institutional Review Boards of Kessler Foundation and Rutgers University Medical School approved the study (which included Experiments 1 and 2), and the study was performed in accordance with the relevant guidelines and regulations. Informed consent was obtained from all subjects.

#### Scan session 1

A 3-Tesla Siemens Allegra scanner was used to acquire all neuroimaging data. Behavioral data acquisition, randomization and stimulus presentation was administered using the E-Prime software^34^. The N-Back paradigm was presented in the scanner in counterbalanced order across participants in an event-related design. A T2*-weighted pulse sequence was used to collect functional images in 32 contiguous slices during eight blocks (four at each of two difficulty levels), resulting in 140 acquisitions per block (echo time = 30 ms; repetition time = 2000 ms; field of view = 22 cm; flip angle = 80°; slice thickness = 4 mm, matrix = 64 × 64, in-plane resolution = 3.438 mm^2^). A high-resolution magnetization prepared rapid gradient echo (MPRAGE) image was also acquired (TE = 4.38 ms; TR = 2000 ms, FOV = 220 mm; flip angle = 8°; slice thickness = 1 mm, NEX = 1, matrix = 256 × 256, in-plane resolution = 0.859 × 0.859 mm), and was used to normalize the functional data into standard space. A fluid attenuation inversion recovery (FLAIR) image was acquired to assess lesion load (TE = 81 ms; TR = 8530 ms; FOV = 256 × 320 mm; flip angle = 180°; slice thickness = 4 mm; matrix = 320 × 154; in-plane resolution = 0.688 × 0.688 mm).

#### Behavioral paradigm

All participants completed a series of practice trials before scanning, exposing them to the two difficulty levels of the N-Back task. During the fMRI scan, participants were presented with the N-Back working memory task in which task difficulty was varied by presenting the 0-back condition, which places a relatively low load on working memory, and the 2-back condition, which places a higher load on working memory. There were 4 blocks of each level of the N-Back task (8 blocks total), with 65 trials per block (16 of which were targets). The 4 blocks of each task were always presented together (that is, the two tasks were not interleaved), and the order of presentation (0-back first vs. 2-back first) was counterbalanced across subjects. During the 0-back task, participants were asked to respond each time the target letter “K” was presented on the screen, while during the 2-back task, participants were asked to respond when the target letter corresponded to the letter presented two trials before. In all cases, the letter stimuli remained on the screen for 1.5 sec. and there was an inter-trial interval of 500 ms.

#### Visual Analog Scale of Fatigue

To evaluate the level of on-task ‘state’ fatigue, participants were presented with a visual analogue scale of fatigue before and after each block of the N-Back task. Participants were asked: “How mentally fatigued are you?” and were asked to indicate their level of fatigue on a scale form 0 to 100, with 0 being not fatigued at all and 100 being extremely fatigued. The visual analogue scale (VAS) has historically been used as a tool for capturing subjective ratings of sensations and feelings or moods. The VAS has been studied with various clinical populations and has demonstrated moderate to high validity and reliability^35^. Previous fMRI studies have utilized the VAS to assess energy and fatigue levels^12, 19^, perceived pain^36^, and changes in mood or emotional intensity^19^. Lee *et al*. developed the VAS-F, a visual analogue scale for capturing fatigue severity that consisted of fatigue and energy subscales^37^. In order to mask the purpose of the study, five additional VASs were administered as well, in randomized order. These assessed happiness, sadness, pain, tension and anger.

#### Questionnaires

After the completion of the fMRI procedure, participants filled out the Fatigue Severity Scale (FSS), Modified Fatigue Impact Scale (MFSI) and Chicago Multiscale Depression Inventory (CMDI). These questionnaires provided a measure of trait fatigue and depression.

### Data analysis

#### Behavioral data

The response time (RT) and accuracy data were each analyzed with linear mixed effects models using the R statistical analysis package (version 1.0.136). The between-subjects factor was Group (TBI vs. HC) and the within-subjects factors were Task (0-back vs. 2-back) and Block (block 1–4 of each task; this was included to account for any order effects, because the four blocks of each task were run sequentially). Three covariates were also included: fatigue, gender and lesion load (see below). The fatigue score used for each block was the average of the VAS-F score reported before and after each block. Because the VAS-F scores differed between the groups, the scores were centered separately for each group prior to analysis. The behavioral data from eight HCs and two individuals with TBI was lost due to equipment failure during scanning (their neuroimaging data is included in the neuroimaging analyses).

The ratings from the visual analog scale of fatigue (VAS-F) were analyzed with a 2 × 4 × 2 mixed, between- and within- subjects ACNOVA, with gender as a covariate. The within-subjects factors were Task (0-back vs. 2-back), and Block (block 1, block 2, block 3, block 4). The between-subjects factor was Group (TBI vs. HC).

#### fMRI data

For each of four fMRI blocks, the first 5 images were discarded to ensure steady state magnetization. All images were preprocessed using Analysis of Functional NeuroImages (AFNI)^38^. The realignment, co-registration and normalization were done in a single transform. This was accomplished by calculating and saving the parameters necessary for realignment using 3dvolreg (i.e., the spatial co-registration of all images in each time-series to the first image of the series). Next, the parameters necessary to co-register the first image in each time-series with the high resolution MPRAGE were calculated and saved (using 3dAllineate). Third, the MPRAGE image (1 × 1 × 1 mm voxels) was warped into standard Talairach space using a non-linear warping algorithm (3dQwarp), and the warping parameters were saved. Finally, the transforms necessary to realign, co-register and warp the data into standard space were combined and applied to the functional time-series data in a single transformation. The images were then smoothed using an 8 × 8 × 8 mm Gaussian smoothing kernel (using 3dBlurToFWHM), and scaled to the mean intensity (using 3dcalc). Each of the four blocks of each task (0-back and 2-back) were then deconvolved separately (using 3dDeconvolve). Motion parameters and two polynomial regressors (to model signal drift) were included as regressors of no interest.

Subject motion: Because it has been shown that clinical populations tend to move more in the MRI scanner than HCs^39^, we analyzed the motion parameters with two between- and within-subjects ANOVAs: one for translational motion and one for rotational motion. For the analysis of translational motion, the between-subjects factor was Group (TBI vs. HC) and the within-subjects factors were Task (0-back vs. 2-back), Run (Run 1–4), and Movement (displacements in the superior/inferior direction, the left/right direction, and the anterior/posterior direction). For the analysis of rotational motion, the factors were the same, but the levels of the factor Movement were roll, pitch and yaw. Both analyses showed a significant effect of Group (translation: F(1,39) = 4.34, p < 0.05; rotation: F(1,39) = 4.95, p < 0.05), and in both cases, this was because the TBI group moved more than the HC group (translation: 0.46 mm for TBIs vs. 0.25 mm for HCs; rotation: 0.45° for TBIs vs. 0.28° for HCs). Because subject motion translates directly into increased noise in the fMRI dataset, these results suggest that the TBI data were noisier than the HC data. In order to control for this, we employed the ‘data scrubbing’ technique proposed by Power *et al*.^40, 41^ in which a ‘framewise displacement’ (FD) is computed for each acquisition. The FD is the sum of the absolute values of the derivatives of the translational and rotational realignment estimates, and is used in two ways: it is included in the deconvolution of each subject’s data as a regressor, and it is used to censor out acquisitions where subjects’ motion was excessive (defined as ≥ 0.5 FD). This was done for all subjects, in both groups, and resulted in the removal of 1.90% of TRs in the HC group and 5.33% of TRs in the TBI group.

Lesion load: Because our sample was moderate/severe TBI, we assessed lesion load in all of our TBI subjects. This was done by manually tracing lesions evident on the FLAIR image using the Jim 6 medical image display package (Xinapse Systems, England). The volume of each lesion was calculated by interpolating across slices; these were then summed to derive total lesion load for each subject^42^.

The fMRI data were analyzed with the same linear mixed effects model as was used for the RT and accuracy data. This model included a between-subjects factor of Group (TBI vs. HC), within-subjects factors of Task (0-back vs. 2-back) and Block (block 1–4 of each task), and the three covariates of fatigue, gender and lesion load. Two follow-up analyses were then run to investigate each group separately. For these analyses, the factors and covariates were the same, with the exception of Group, which was omitted. All group-level statistical maps were thresholded using both the alpha level and cluster size correction (extent of activation). The alpha level was set at p < 0.01 and the cluster size was set at 108 contiguous voxels. The results of Monte Carlo simulations showed that this combination resulted in a corrected alpha level of p < 0.05. Because we had a prior hypothesis about the involvement of the caudate, a separate Monte Carlo simulation was conducted on just the caudate. Based on this, clusters of at least 26 voxels within the caudate were also considered significant.

## Results

### Behavioral results

For RT, as expected, there were main effects of both Group (F(1,21.1) = 4.66, p < 0.05) and Task (F(1,150.9) = 94.42, p < 0.0001). The effect of Group resulted from individuals in the TBI group responding with longer latencies (RT = 742 ms) than individuals in the HC group (RT = 665 ms). The effect of Task resulted from subjects responding with longer latencies when performing the 2-back task (RT = 779 ms) than when performing the 0-back task (RT = 643 ms). The interaction between Group and Task was not significant.

For the accuracy data, as expected, there was a main effect of Task (F(1,159.4) = 73.67, p < 0.0001). This resulted from subjects being significantly less accurate on the 2-back task (87.9%) compared to the 0-back task (96.4%). Task also interacted with Fatigue (F(1,161.0) = 8.37, p < 0.005), which was due to a stronger relationship between fatigue scores and accuracy during the 2-back task (coefficient = 0.0017) than between fatigue scores and accuracy during the 0-back task (coefficient = 0.00004). Finally, there was an interaction between Group, Task and Fatigue (F(1,162.9) = 4.32, p < 0.05). This resulted from the interaction between Task and Fatigue (above) being seen mainly in the HC group. Thus, the relationship between fatigue and accuracy for the 2-back task in the HC group was stronger (coefficient = 0.0013) than for the 0-back task (coefficient = 0.00008). For the TBI group, the relationship between fatigue and accuracy was relatively weak for both tasks (0-back coefficient = −0.00050; 2-back coefficient = 0.00026).

### VAS results

The main effect of Group was significant (F(1,46) = 7.51, p < 0.01, η^2^ = 0.12), which resulted from the TBI group reporting greater fatigue (35.8) than the HC group (16.8). The main effect of Block was also significant (F(4,184) = 6.48, p < 0.001, η^2^ = 0.01), which resulted from subjects reporting more fatigue as the experiment (blocks) progressed (VAS scores = 22.9, 26.1, 26.4, 29.7, 30.7). No other main effects or interactions were significant.

### fMRI results

In the analysis of BOLD data, there were main effects of Group and of Task, and there were Group X Task interactions (see Table 1). These effects, which do not involve fatigue, are consistent with the literature on working memory in TBI; they are reported here for completeness, but because fatigue is the primary focus of the current paper, effects that do not involve fatigue are not discussed further.

**Table 1:** Areas of significant brain activation for the analysis of the N-Back task.

Analysis of N-Back task						
	*BA*	X	Y	Z	Voxels	F Stat.
**Group**						
Cingulate Gyrus	*32*	11	9	36	126	20.83
**Task**						
Superior Frontal Gyrus	*8*	−13	41	44	223	17.97
Superior Frontal Gyrus	*10*	25	47	4	119	14.7
Middle Frontal Gyrus	*6*	−27	−3	56	23538	92.56
Parahippocampal Gyrus	*36*	−31	−33	−8	233	15.62
Cuneus	*18*	13	−75	6	219	14.3
Lingual Gyrus	*18*	−15	−73	0	121	13.42
**Group x Task**						
Middle Frontal Gyrus	*9*	43	19	32	547	20.58
Angular Gyrus	*39*	37	−55	32	319	15.54
Parahippocampal Gyrus	*36*	39	−31	−14	168	17.29
**Group x Fatigue**						
Precuneus	*31*	3	−69	28	150	11.55
Lingual Gyrus	*18*	−33	−71	−8	191	32.87
**Task x Fatigue**						
Middle Frontal Gyrus (extending into medial PFC and caudate head)	*6*	−25	−11	54	4165	26.25
Middle Frontal Gyrus	*6*	25	−5	52	181	24.08
Inferior Frontal Gyrus	*9*	49	−1	24	899	27.9
Inferior Frontal Gyrus	*10*	33	37	8	288	21.89
Caudate/Caudate Head	*—*	1	7	4	46	10.85
Cingulate Gyrus	*31*	−15	−35	42	295	16.53
Precentral Gyrus	*6/9*	−51	−1	24	1599	26.47
Inferior Parietal Lobule	*7*	−29	−53	40	1855	42.34
Inferior Parietal Lobule	*40*	33	−51	40	1213	24.28
Suprior Temporal Gyrus	*41*	45	−31	12	465	17.36
Parahippocampal Gyrus	*30*	−17	−37	6	141	12.32
Cuneus	*18*	13	−83	24	133	14.96
Fusiform Gyrus	*19*	−33	−73	−12	715	20.06
Middle Occipital Gyrus	*19*	37	−79	4	135	17.59
**Group x Task x Fatigue**						
Middle Frontal Gyrus	*9*	−39	35	36	312	33.66
Middle/Superior Frontal Gyrus	*9/10*	37	37	26	221	12.88
Medial Fronal Gyrus	*8*	1	27	38	348	16.13
Inferior Frontal Gyrus	*10*	−35	37	4	561	18.48
Caudate/Caudate Body	*—*	−1	7	6	61	11.96
Precentral Gyrus	*9/6*	45	−1	26	407	24.17
Precentral Gyrus	*6*	−47	−1	48	213	25.42
Angular Gyrus	*39*	−29	−55	38	461	22.91
Superior Temporal Gyrus	*22*	−53	−35	6	318	17.84
Superior Temporal Gyrus	*22*	53	−27	2	228	17.39
Middle Occipital Gyrus	*19*	−37	−77	−10	333	22.81
Fusiform Gyrus	*19*	31	−75	−16	475	24.17
Declive	*—*	−35	−65	−18	252	15.99

‘BA’ denotes Broadman’s Area; ‘X’, ‘Y’, and ‘Z’ denote the location in Talairach space of the voxel in the cluster with the highest activation; ‘Voxels’ denotes the number of voxels in each cluster; ‘F-Stat.’ denotes the F-statistic associated with the voxel of highest activation.

As Table 1 shows, there was a Group X Fatigue interaction in the precuneus and lingual gyrus. The interaction in the precuneus resulted from HCs showing a negative relationship between fatigue scores and activation in the precuneus (coefficient = −0.0021) while the TBI group showed a positive relationship (coefficient = 0.00076). That is, those individuals with TBI who reported higher fatigue had more activation in the precuneus while HCs who reported lower fatigue had more activation in the precuneus. This pattern was reversed in the lingual gyrus where HCs showed a positive relationship between fatigue scores and activation (coefficient = 0.0011) and the TBI group showed a negative relationship (coefficient = −0.00068). Taken together, these results show that in the TBI group, lower fatigue was associated with lower activation in the precuneus and greater activation in visual processing areas (lingual gyrus) whereas in the HCs lower fatigue was associated with greater activation in precuneus and lower activation in visual processing areas (lingual gyrus).

Fatigue interacted with Task in several areas (collapsing across Group). These included frontal areas (middle and inferior frontal gyri, and the cingulate), basal ganglia (caudate), inferior parietal areas, temporal areas (superior temporal gyrus and parahippocampal areas), and occipital areas (cuneus, fusiform gyrus and middle occipital gyrus). (see Table 1). Because there was no interaction with Group in these areas, they are included for completeness.

**Figure 2:**
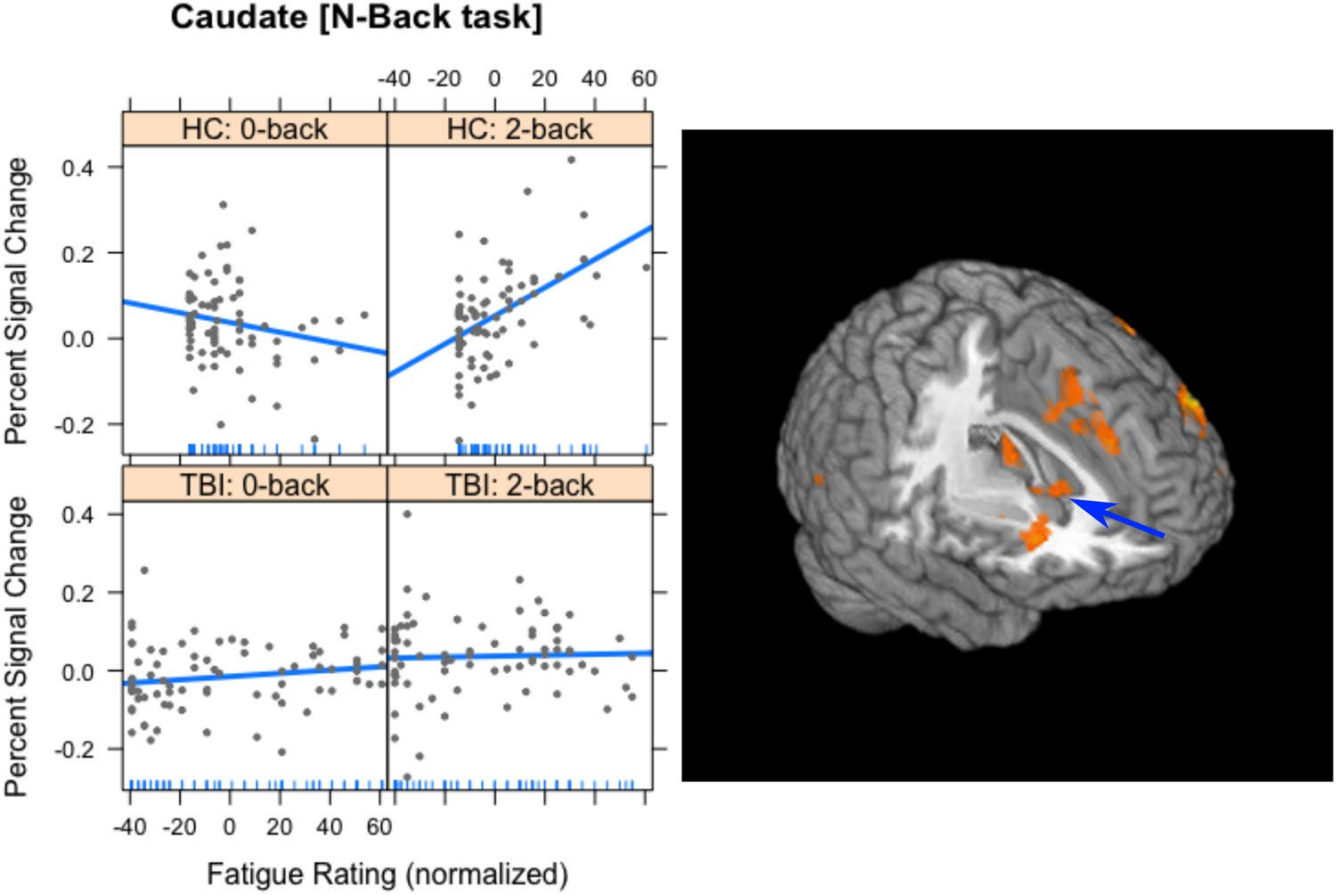
The interaction of Group, Task and Fatigue in Experiment 1 in the caudate head (indicated by blue arrow). The plots on the left are included only to show the slopes of the regression lines. For the HC group, there was a negative correlation between fatigue and activation for the 0-back task (top left), and a positive correlation for the 2-back task (top right). For the TBI group, the correlations between fatigue and activation in both tasks were very weak (lower plots). In all cases, the vertical axis is the percent signal change in the caudate head and the horizontal axis is the normalized fatigue score. On the right is a 3-dimensional rendering of the activation in the caudate head for Experiment 1.

Finally, there were several regions where Fatigue interacted with Group and Task. These are detailed in Table 1 and included frontal areas (superior, middle, inferior and medial gyri), basal ganglia (caudate), temporal areas (superior temporal gyri), occipital areas (middle occipital and fusiform gyri) and cerebellum (declive). Because we had prior hypotheses about the caudate, the differences in activation there where investigated further (see Fig. 2). This showed that for the HC group, the activation in the caudate was negatively associated with fatigue during the 0-back task (coefficient = −0.0013); however, during the 2-back task, the relationship between activation and fatigue was positive (coefficient = 0.0035). This pattern was not evident in the TBI group. Indeed, for the TBI group, the relationship between fatigue and activation during either task was very weak (coefficients = 0.0003 for 0-back and 0.0002 for 2-back).

### Group-specific results

In order to better understand the relationship between fatigue and brain activation following a TBI, we performed two more analyses in which we focused on the data from each group separately. The factors were the same as in the analysis above, with the exception of Group, which was omitted.

**Figure 3:**
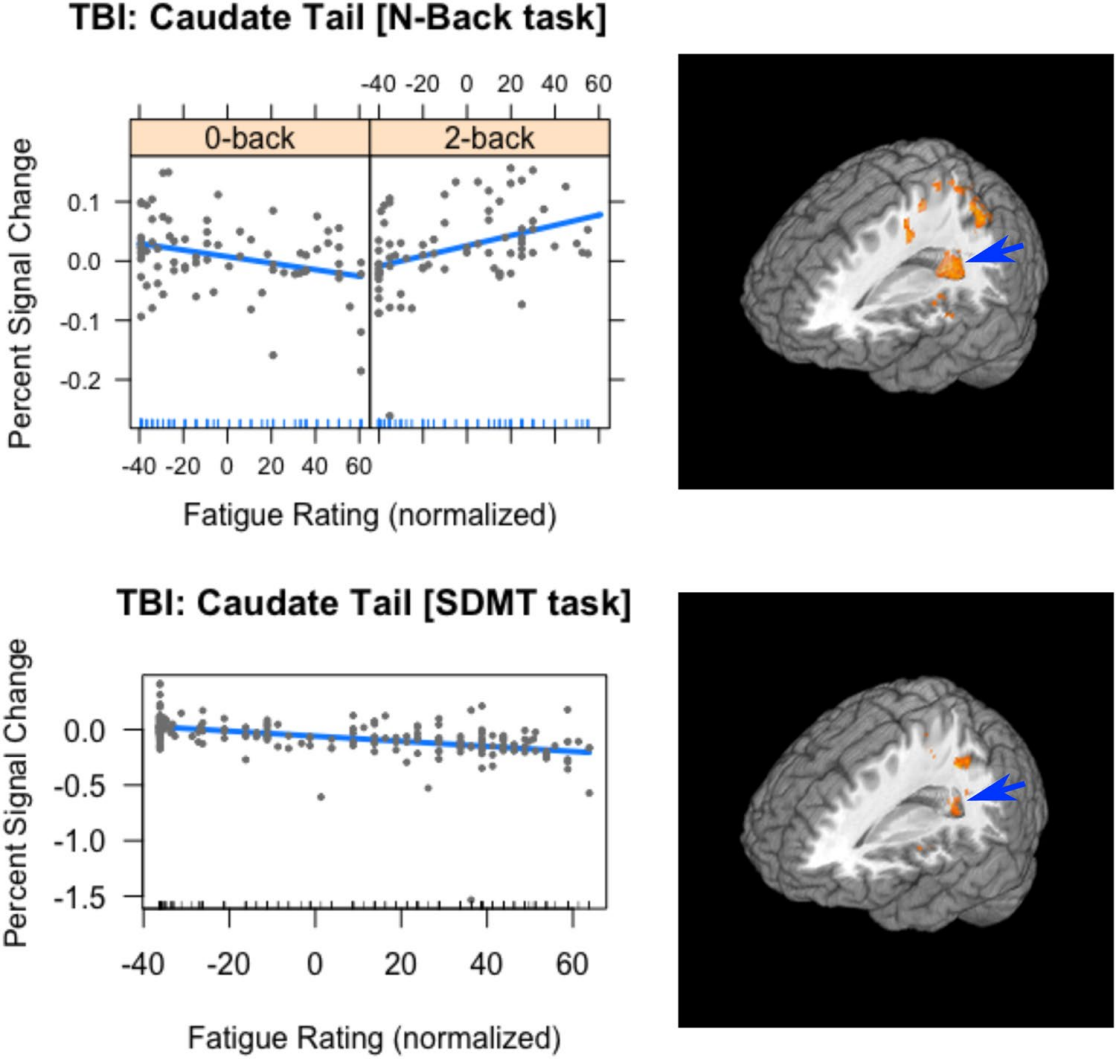
The relationship between Fatigue and activation in the caudate tail in the TBI group across Experiments. The plots on the left are included only to show the slopes of the regression lines. For the 0-back task (top left), there was a negative relationship between Fatigue and activation, while the relationship was positive for the 2-back task (top right). For the SDMT, the relationship between Fatigue and activation was not modulated by Task: for both tasks the relationship was negative (lower plot). For all plots the vertical axis is the percent signal change in the caudate tail and the horizontal axis is the normalized fatigue score. On the right are 3-dimensional renderings of the activation in the caudate tail (indicated by blue arrow) for the N-Back task (top) and the SDMT (bottom).

For the TBI group, there was a main effect of Fatigue in the caudate (coefficient = 0.00054), the posterior cingulate (coefficient = 0.0082) and in the superior temporal gyrus (coefficient = 0.00076) (see Table 2). All three coefficients were positive, meaning that as activation increased in these areas, fatigue increased as well. Furthermore, Fatigue interacted with Task in frontal areas (the anterior cingulate and insula), in the basal ganglia (caudate), the posterior cingulate bilaterally, and in parietal areas (the precuneus and inferior parietal areas). As Fig. 3 shows, the activation in the caudate was due to a negative relationship between fatigue and activation during the 0-back task (coefficient = −0.0005), whereas during the 2-back task the relationship between fatigue and brain activation was positive (coefficient = 0.0009). This interaction is similar to the interaction seen in the HC group in the head of the caudate (see Fig. 2), but was more posterior.

**Table 2:** Areas of significant brain activation for the analysis the N-Back task, separately for each Group.

Analysis of N-Back task, separated by Group								
			*BA*	X	Y	Z	Voxels	F Stat.
	**Fatigue**							
Group: TBI	Caudate/Caudate Tail		—	−19	−25	16	36	12.96
	Posterior Cingulate		*23*	9	−39	24	247	16.32
	Superior Temporal Gyrus		*39*	−41	−51	12	109	17.8
	**Task x Fatigue**							
	Anterior Cingulate		*24*	−7	31	6	265	19.39
	Caudate/Caudate Body		—	13	−13	22	286	18.38
	Caudate Tail/Posterior Cingulate		*30*	−21	−47	14	236	15.33
	Insula		*13*	−37	−25	18	273	21.75
	Precentral Gyrus		*4*	−19	−21	56	702	17.52
	Posterior Cingulate		*30*	23	−61	12	156	13.54
	Precuneus		*7*	27	−47	42	371	17.67
	Inferior Parietal Lobule		*40*	39	−33	38	343	22.81
	**Task X Fatigue**							
Group: HC	Middle Frontal Gyrus		*10*	37	43	24	345	17.62
	Middle Frontal Gyrus		*6*	−27	−5	58	312	8.15
	Middle Frontal Gyrus		*6*	23	1	50	249	7.29
	Medial/Superior Frontal Gyrus		*10*	−19	45	2	2053	9.02
	Medial Frontal Gyrus, extending into right caudate head		*32*	−9	13	44	1756	8.73
	Inferior Frontal Gyrus		*9*	59	9	28	635	7.69
	Caudate/Caudate Head		—	3	1	6	564	11.46
	Caudate/Caudate Tail		—	−15	−31	20	36	9.95
	Thalamus/Pulvinar		—	−25	−25	4	125	7.9
	Thalamus		—	7	−23	2	219	13.63
	Insula		*13*	−43	−5	18	776	7.11
	Insula		*13/22*	41	−27	2	567	7.76
	Precental Gyrus		*6*	−43	−7	44	437	7.55
	Inferior Parietal Lobule		*40*	37	−39	48	558	7.77
	Middle Occipital Gyrus		*19/37*	43	−71	4	382	8.85
	Fusiform Gyrus		*37*	37	−53	−16	191	12.72
	Lingual Gyrus		*18*	−31	−71	−10	2154	14.24

‘BA’ denotes Broadman’s Area; ‘X’, ‘Y’, and ‘Z’ denote the location in Talairach space of the voxel in the cluster with the highest activation; ‘Voxels’ denotes the number of voxels in each cluster; ‘F-Stat.’ denotes the F-statistic associated with the voxel of highest activation.

For the HC group, Fatigue interacted with Task in several frontal areas (middle, medial and inferior frontal gyri, and the insula bilaterally), the basal ganglia (caudate), the thalamus, inferior parietal areas, and occipital areas (middle occipital, fusiform and lingual gyri) (see Table 2).

## Experiment 2

In Experiment 2, subjects were asked to perform the modified symbol-digit modalities task (SDMT), which is a task that relies heavily on processing speed^8^, particularly in the visual domain. In this task, subjects must match a probe stimulus against a list of potentially matching stimuli to see whether the probe does or does not match. This task thus is very different from the N-Back task in Experiment 1, and was included as a test for the generalizability of the findings from Experiment 1. We reasoned that if the caudate is implicated in fatigue, we should find fatigue-related activation in the caudate regardless of the task.

### Methods

The subjects, VAS and data preprocessing were the same as in Experiment 1. The scanning parameters were comparable, with the single difference that 240 volumes of functional data were acquired because each block of the SDMT task was longer than the N-Back blocks.

#### Behavioral Paradigm

On each trial, subjects were presented with a reference grid at the top of the screen that had two rows and nine columns^8^. In the top row were the numbers 1–9, in the bottom row were nine unique symbols. Below this reference grid a probe stimulus was presented that consisted of one number and one symbol. For the SDMT task, subjects reported whether the probe number-symbol pair matched the number-symbol pair in the reference grid. As with the N-Back task, a control task was also included, which was similar in stimulation, but was far easier to perform. Here, the control task consisted of the same stimuli, but when there was a match between the probe and the reference grid, it was highlighted by a yellow box overlaid on the reference grid. Thus, as with the 0-Back condition of the N-Back task, the control condition was essentially a visual monitoring task.

#### Data processing

The analyses of the behavioral data and the VAS data were the same as for Experiment 1. For the fMRI data, we restricted the analysis to the caudate, since our *a priori* hypothesis was specific to the caudate. Moreover, because the analysis of the N-Back task had showed that the areas in the caudate associated with fatigue were different in the TBI and HC groups, we confined all analyses to the TBI group.

## Results

### Behavioral results

For RT, there was a main effect of Task (F(1,95.79) = 1313.00, p < 0.0001), resulting from individuals in the TBI group responding with longer latencies during the SDMT task (RT = 2004.1) than during the control task (RT = 936.0). The effect of Fatigue approached significance (F(1,107.58) = 3.02, p = 0.08), resulting from a negative relationship between Fatigue and RT (coefficient = −0.70). That is, the longer their latencies, the less fatigue they reported. Finally, there was an interaction between Task and Fatigue (F(1,96.65,p < 0.001) resulting from a negative relationship between RT and Fatigue for the SDMT task (coefficient = −0.41), and a positive relationship for the control task (coefficient = 0.95).

In the accuracy data, there was a significant main effect of Task (F(1,96.09) = 22.55, p < 0.0001): subjects were less accurate on the SDMT task (93.4%) than the control task (99.4%). The only other effect to approach significance was that of Fatigue (F(1,58.15) = 3.21, p = 0.08). This was due to a positive relationship between fatigue and accuracy (coefficient = 0.0006): the more accurate individuals with TBI were, the more fatigue they reported.

### VAS results

The main effect of Group was significant (F(1,46) = 7.03, p < 0.001, η^2^ = 0.11), which resulted from individuals in the TBI group reporting more fatigue (36.1) than individuals in the HC group (14.4). The effect of Block was also significant (F(4,184) = 12.25, p < 0.001, η^2^ = 0.01), which resulted from subjects reporting more fatigue as the experiment (blocks) progressed (VAS scores = 23.0, 27.4, 27.4, 31.4, 33.1).

### fMRI results

In this confirmatory analysis, only voxels anatomically defined within the caudate were included. The same thresholding criterion was used as above. As Fig. 3 shows, there was a main effect of Fatigue in the caudate (X Y Z = −17, −41 12; extent = 26 voxels; F = 12.18). Unlike in Experiment 1, there was no interaction between Task and Fatigue. Rather, there was a negative relationship between Fatigue and activation in the caudate across Task in the TBI group (coefficient = −0.0023).

## Discussion

The aim of the current study was to understand the neural correlates of cognitive fatigue in individuals who have sustained a TBI. In Experiment 1, we used a working memory task to induce fatigue because prior studies have shown that individuals with TBI experience deficits in working memory^43–45^. In Experiment 2, we used a task that relies on processing speed to induce fatigue, as we have used in the past^8^. The results from both studies showed a relationship not only between subjective cognitive fatigue and activation in brain areas such as the caudate, but also between subjective cognitive fatigue and behavioral performance. For the N-Back task, there was a three-way interaction between Group, Task and Fatigue in the accuracy data, such that as accuracy increased during the 2-back task, fatigue also increased. This relationship was far weaker for the 0-back task, and was weak for both tasks in the TBI group. Subjects’ accuracy during the 2-back task suggests that the task was difficult for them (87.9% correct during the 2-back compared to 96.4% during the 0-back task), and the positive relationship between fatigue and accuracy during the 2-back suggests that as subjects worked harder to improve their accuracy, cognitive fatigue resulted.

The differential effect of the two tasks on fatigue was also evident in the brain activation data for the N-Back task. Activation in the head of the caudate showed a pattern very similar to that seen in the accuracy data: a three-way interaction between Group, Task and Fatigue. As activation increased during the 2-back task, fatigue also increased (similarly to the accuracy data). During the 0-back task, this positive correlation was not evident; indeed, there was a negative correlation such that as activation increased, fatigue decreased. Finally, as with the accuracy data, this relationship was seen in the HC data and was essentially absent in the data from the TBI group. However, when the data from the TBI group was analyzed separately, a qualitatively similar interaction was evident in the caudate tail: a positive relationship between fatigue and activation for the 2-back task, and a negative relationship for the 0-back task.

For the SDMT task, there was also an interaction between activation in the caudate tail and self-reported fatigue. However, in this case the behavioral data and the functional imaging data diverged. The RT and accuracy data for the SDMT showed that individuals with TBI found the SDMT to be more difficult than the control task: they responded with longer latencies and made more errors during the SDMT than during the control task. However, while the activation in the caudate tail was related to fatigue, it was not modulated by task. Moreover, the relationship between activation in the caudate tail and fatigue for the TBI group was negative, meaning that increased activation in the caudate tail was associated with decreased fatigue. This was also the case during the less difficult condition of the N-Back task (0-back).

The negative relationships between activation in the caudate and self-reported fatigue are consistent with the Dopamine Imbalance Hypothesis^46–48^ that suggests a U-shaped relationship between the amount of dopamine in the brain (or activation in the structures enervated by dopamine) and levels of fatigue. That is, at low levels of dopamine, fatigue is high; as levels of dopamine increase, fatigue decreases; however, at high levels of dopamine, fatigue increases again. Indeed, it has been shown that dopamine agonists (e.g., methylphenidate) lead to fatigue reduction in individuals with TBI^49^ and other clinical populations^50, 51^. The caudate nucleus is one of the structures enervated with dopamine. Dopaminergic activation of the striatum and firing of dopamine neurons has been shown to be crucial in reward processing, as well as during working memory tasks such as the N-Back and during attentional tasks such as the SDMT. For example, several studies have shown a correlation between dopamine release and working memory capacity^52, 53^. The Dopamine Imbalance Hypothesis proposes that dopamine levels are disrupted following brain injury or disease, due to damage of frontal dopaminergic systems. Additionally, brain injury such as a TBI also disrupts information transmission (due to diffuse axonal injury [DIA]) resulting in less efficient, more effortful processing^54^. Taken together, the increase in the effort required to perform cognitive work following a TBI creates an imbalance between effort and outcome. It is our larger hypothesis that fatigue is a signal the brain generates when the outcome (or reward) no longer merits the effort required^30, 55^. On this view, low dopamine and DAI work together to change the balance between effort and reward in TBI, resulting in persistent fatigue.

For the SDMT, the SDMT control task, and the 0-back task, lower activation in the caudate was associated with higher self-reported fatigue, which is consistent with the Dopamine Imbalance Hypothesis. However, for the 2-back task, the relationship between activation in the caudate and fatigue was reversed: more activation was associated with more fatigue. This too is consistent with the Dopamine Imbalance Hypothesis, because the relationship between dopamine levels and fatigue is proposed to have a U-shaped function^30^. Thus, while low levels of dopamine result in fatigue, so too do high levels of dopamine. Because the 2-back task is a challenging working memory task that requires frontal circuits, continued performance of the 2-back likely results in increased release of dopamine, and thus in increased fatigue. This is true for both groups, though in different areas of the caudate. For the less challenging 0-back task, and for both the SDMT and the SDMT control task, which are more visual tasks, there is less frontal involvement, leading to less dopamine release. Because the relationship between dopamine and fatigue is proposed to have a U-shaped function, this can also lead to fatigue.

As previous attempts to study cognitive fatigue using ‘trait’ measures (i.e., FSS) have been inconsistent, we used a measure of ‘state’ fatigue: the VAS-F. In Experiment 1, individuals with TBI reported more cognitive fatigue on the N-Back task than HCs, even when the task demands were low (0-back). Furthermore, for both groups, self-reported fatigue was found to covary with brain activation in the caudate: the caudate head in HCs and the caudate tail in individuals with TBI. The involvement of the caudate with fatigue replicates and extends previous work that has investigated fatigue in several clinical populations, including individuals with TBI^8, 9, 18^, multiple sclerosis^19, 56, 57^ and healthy controls^58^. For example, in Kohl *et al*.^8^, we showed that activation in the caudate nucleus differed between HCs and individuals with TBI during continued performance of the SDMT. In that study, we operationally defined a difference between HCs and individuals with TBI to be fatigue, but we could not verify this definition in our sample because no measure of subjective fatigue was collected. Here we have shown that the relationship between subjective cognitive fatigue and caudate activation is negative in the TBI group for three tasks that do not heavily rely on frontal systems (the 0-back task, the SDMT and the control task for the SDMT), such that increased activation in the caudate is associated with lower self-reported fatigue. For a task that does rely on frontal systems (the 2-back task), the relationship between subjective cognitive fatigue and caudate activation is positive, which we interpret as representing the higher-dopamine arm of the U-shaped function describing the relationship between dopamine and fatigue. These findings reinforce the importance of the role of the caudate in cognitive fatigue^17^.

It was also expected that, as the task demands were increased, the TBI group would report greater fatigue. The results did not support this expectation in either experiment. In fact, while self-reported fatigue (VAS-F) increased in both groups throughout task performance, this increase did not vary by task for either group, and this was true in both experiments. These findings accord well with recent work in MS, in which we found that fatigue increased as a function of time-on-task, rather than as a function of task difficulty^21^. The similarity in the subjective reports of fatigue across these two populations (MS and TBI) suggests that cognitive fatigue, assessed following task performance, may rely on similar mechanisms across these two clinical populations.

Another similarity across the TBI and MS populations is that state fatigue measures were associated with activation in the caudate. While there is no evidence that the caudate was itself damaged in any of the individuals in our TBI group, there is evidence from animal studies that brain trauma in rats results in decreased dopamine transmission in the caudate^31^. Therefore, our finding that the caudate responds differently to fatigue in individuals who have sustained a TBI relative to controls may reflect differences in dopamine transmission in the two groups. Indeed, because the basal ganglia are strongly dopaminergic, these observations suggest that the basal ganglia, and the caudate in particular, may represent a target for clinical interventions to alleviate fatigue in TBI. Indeed, it has been shown that dopamine agonists (methylphenidate) reduce fatigue in individuals with TBI^49^, a result that is entirely consistent with our findings.

There are, of course, some limitations to the conclusions we can draw from these data. The sample size was relatively modest, and it would be encouraging to see these results replicated in a larger sample. Moreover, because the design of the current study is cross-sectional, we cannot conclude that increased activation of the caudate causes decreased fatigue in individuals with TBI for tasks that do not rely on frontal systems. While it is tempting to make assertions of this kind, longitudinal studies are required to draw conclusions regarding causation. Finally, the analyses here rely on a self-report measure of fatigue, the VAS-F, and it cannot be determined with absolute certainty that all subjects rated their fatigue in exactly the same way. For example, some subjects may have reported ‘fatigue’ because they were bored while others may have reported ‘fatigue’ because they had worked hard on the mental tasks, and it is therefore difficult to determine whether subjects’ reports of ‘fatigue’ derived from boredom or mental labor. This concern goes to the heart of the nature of fatigue. It is our view that fatigue is a signal the brain generates to ‘tell’ itself that sufficient time and energy have been expended on the task at hand and that it is time to stop performing that task. On this view, fatigue is an outcome that derives from a number of potential causes, including labor, boredom, depression, etc. It may be valuable to study fatigue that derives from each potential source (e.g., labor, boredom, depression, etc.), but the results reported here suggest that – whatever the source – the experience of fatigue appears to be associated with activation in the caudate nucleus of the basal ganglia.

## Conclusions

In this work, we have demonstrated that fatigue-related brain activation can be induced and measured in TBI. This extends our prior work using a similar approach in MS^19^, and adds to the work that has been done investigating the neural correlates of fatigue in TBI^8, 59^. The results reported here underscore the importance of the caudate nucleus in relation to cognitive fatigue. Furthermore, the results reported here also show that levels of self-reported state fatigue do not vary as a function of task difficulty, which is in line with recent work in MS^21^.
